# Predictors of serological cure after penicillin therapy in HIV-negative patients with early syphilis in Shenzhen, China

**DOI:** 10.1371/journal.pone.0245812

**Published:** 2021-01-28

**Authors:** Zhenzhou Luo, Yi Ding, Jun Yuan, Lishan Tian, Li Zhang, Qiuhong Wu, Jinsong Mou

**Affiliations:** 1 Shenzhen Nanshan Center for Chronic Disease Control, Shenzhen, China; 2 Shenzhen Pingshan Maternal and Child Health Hospital, Shenzhen, China; The Chinese University of Hong Kong, HONG KONG

## Abstract

**Background:**

Syphilis is a common infectious disease worldwide. Serological monitoring is important for syphilis management. We currently know little about the characteristics of this seronegative response. The aim of this study was to explore the factors associated with serological cure after treatment of early syphilis.

**Methods:**

A retrospective cohort study was conducted and the data of patients with early syphilis in a clinic in Shenzhen from 2011 to 2019 were retrieved. Univariable and multiple Cox proportional hazard regression models were utilized to identify factors associated with a serological cure state among syphilis patients with early syphilis two years after treatment.

**Results:**

A total of 346 (85.9%) syphilis patients achieved serological cure. The multivariate analysis results revealed that having a baseline TRUST titer >1:8 was associated with an increased probability of serological cure, compared with having a baseline TRUST titer ≤1:8 (HR = 1.43, 95% CI = 1.10–1.85, P<0.01); primary syphilis was positively associated with serological cure, compared with participants with latent early syphilis (HR = 1.72, 95% CI = 1.27–2.33, P<0.001).

**Conclusions:**

Two years after treatment, a higher percentage of early syphilis patients achieved serological cure. The study indicated that the syphilis stage and baseline serum titer were crucial factors associated with serological cure.

## Introduction

Syphilis is a sexually transmitted disease caused by *Treponema pallidum*, and is spread mainly through direct lesion contact [1]. The disease can progress over years without treatment through a series of clinical stages and lead to irreversible neurological or cardiovascular complications.

Although Treponema pallidum can be treated easily and inexpensively with antibiotics [2], syphilis remains a prevalent disease worldwide. More than 5 million new cases of syphilis are diagnosed every year in the world [3,4]. In China, syphilis has imposed an increasing burden in the past decade. The average syphilis incidence showed significantly increasing trends from 2004 to 2013 and fast rates of growth with annual percentage changes of 16.3% [5]. In 2018, the syphilis incidence increased to 35.6 cases per 100,000 [6].

Recent reports on the incidence of syphilis in Guangdong Province and Shenzhen District reported 52.55 cases [7] and 64.04 cases [8] per 100,000 people, respectively. Shenzhen, which is located in Guangdong Province of southern China, just north of Hong Kong, is the first special economic zone in China. However, in Shenzhen, syphilis showed a significant rise and an obvious spatial distribution [9–11]. The burden of syphilis in Shenzhen was large and syphilis control should be regarded as a public health priority.

Some papers have examined the factors associated with the successful treatment of syphilis. The results of these studies showed that serological cure was associated with age, sex, sex partners, baseline serological titers, syphilis stage and fluorescent treponemal antibody absorption (FTA-ABS) IgM [12–14]. The identified factors associated with serological response to syphilis treatment have implications for the early management of syphilis and expected outcomes after therapy.

To consolidate prevention, treatment and management to better control syphilis, in April 2011, Nanshan District in Shenzhen City, launched the Syphilis Convergence Case-management Program. All the health organizations in the district were asked to refer seropositive syphilis cases to the STD clinic, Department of Dermatology and Venereology in Nanshan Center for Chronic Disease Control, which provided centralized management, standardized treatment and regular serologic follow-up. In the process of running the program, benzathine penicillin G (BPG) is recommended for early syphilis treatment, which refers to latent early syphilis, primary syphilis and secondary syphilis infections with Treponema pallidum within 2 years and patients’ response to therapy is assessed based on changes over months of serological test titers [15,16].

Serological assays to quantitatively detect nontreponemal antibodies are the mainstay for evaluating the effectiveness of treatment. The toluidine red unheated serum test (TRUST) is a procedure that labels the antigen with toluidine red particles [17], which is mostly used in serum tests for laboratory diagnosis of syphilis. Correct understanding and appropriate interpretation of the serological response following therapy are critical to guide clinical practice regarding the needs for additional follow-ups or therapy. However, we still know little about the serological response and the associated factors that predict the serological response after syphilis treatment have not been well defined in this area. As immune differences and HIV drugs have some effects on the outcome of HIV-positive and HIV-negative syphilis patients, the aim of this study was to estimate the rates of serological cure after treatment with BPG and identify the factors related to the serological response among HIV-negative patients with early syphilis.

## Methods

### Study population

We performed a retrospective cohort study and data from patients who were diagnosed with early syphilis in our clinic between 2011 and 2019 were obtained in August 2020 with application to syphilis studies. All patients were told that their medical records would be used for research when they were recruited in the program and received the first treatment. Informed written consent was obtained from the patients who agreed to participate in this study, and their participation was voluntary. The patients were recruited into the study when they met the following criteria: (1) being diagnosed with early syphilis; (2) having enough clinical records, such as demographical characteristics, baseline TRUST titer and disease stages; (3) not having an allergy to penicillin after receiving treatment with benzathine penicillin G (BPG); (4) being negative for human immunodeficiency virus (HIV); and (5) completing at least one serological follow-up visit after treatment. Simultaneously, patients with the following conditions were excluded: severe heart, liver and kidney diseases; malignant diseases; chronic infectious diseases, such as tuberculosis, leprosy, and viral hepatitis; systemic autoimmune diseases, such as lupus erythematosus, rheumatoid arthritis and dermatomyositis; and severe mental illness.

### Diagnosis, treatment and follow-up

Early syphilis diagnosis, treatment and follow-up were in compliance with the 2014 national guidelines in China [16]. The patients were finally diagnosed with early syphilis according to their epidemiological history, clinical signs and symptoms, and TRUST/TPPA results. Non–penicillin-allergic participants without HIV were treated with BPG in one course (BPG 2.4 million units weekly for 2 weeks as one course). Follow-up was initiated after treatment, and nontreponemal test titers were obtained to evaluate patients’ serological response at 3, 6, 9 and 12 months in the first years and 18 and 24 months in the second year.

### Laboratory test

Blood specimens were tested for syphilis. The toluidine red unheated serum test (TRUST, Rongsheng Biotech Company, Shanghai, China) and *Treponema pallidum* particle agglutination test (TPPA, Fujirebio, Tokyo, Japan) were performed on serum samples. The operational instructions were strictly obeyed and the outcomes were evaluated by two researchers.

### Definition of serological cure

The primary outcome was the response to therapy, as determined by the changes in the TRUST titers after treatment. All the participants were recruited in the cohort and were followed periodically. Serological cure was defined as either a negative TRUST test or ≥4 fold decreased titer after treatment during follow-up.

### Statistical analysis

Patient information was recorded in a database using Excel and was analyzed using Stata16. Descriptive statistics were used to describe the baseline characteristics and the serological conversion status. The endpoint was defined as the outcome of the TRUST test becoming negative or a ≥4 fold decreased titer. If the endpoint was not reached at the end of the study, then the patient was defined as censored. If someone was lost to follow up, then the final follow-up result was used. Univariable and multivariate Cox proportional hazard analyses were performed to analyze the predictors of serological cure after treatment. The p value equal to or less than 0.05 was considered as statistically significant.

### Ethics approval and consent to participate

This study was approved by the Institutional Ethics Committee of Nanshan Center for Chronic Disease Control and was in compliance with the national legislation and the Declaration of Helsinki guidelines (LL20200001). Informed written consent was obtained from the participants who agreed to participate in this study, and their participation was voluntary.

## Results

### Characteristics of the study population

A total of 1280 syphilis patients were screened, and 862 patients who did not meet the inclusion criteria, such as those who did not receive regular treatment, had latent syphilis, were HIV-positive and had missing data, were excluded from the analysis. Twenty patients were lost to follow up because of a change in residence or lost communication. A total of 418 eligible patients were finally included in the analysis. A total of 298 (71.3%) and 120 (28.7%) patients were male and female, respectively, resulting in a sex ratio of 2.5:1. The average patient age was 33.5±11.0 years, and the ages ranged between 17 and 77 years old. A total of 230 (55.0%) and 188 (45.0%) were unmarried and married, respectively. A total of 136 (32.5%) patients had baseline TRUST titers of ≤1:8, and 282(67.5) patients had baseline TRUST titers of >1:8. A total of 149 (35.7%), 100 (23.9%), and 169 (40.4%) patients were diagnosed with latent early syphilis, primary syphilis and secondary syphilis, respectively, and 391 (95.4%) patients were treated with one course of BPG. Fifty-seven (13.6%) patients were male homosexual and 15 (3.6%) patients were bisexual. Forty-two (10%) patients received the syphilis test passively, and 376 (90%) patients received the syphilis test actively.

### Serological response to therapy

All the nonpenicillin-allergic participants received BPG. The proportions of evaluable participants who exhibited serological cure varied by syphilis stage and time point after therapy. Overall, the proportion of serological cure at 3, 6, 9, 12, 18, and 24 months separately were 72.25% (302/418), 79.43% (332/418), 82.1% (343/418), 84.9% (355/418), 85.2% (356/418) and 85.9% (359/418), respectively. The proportion of serological cure significantly increased over the time of follow-up (chi-square for trend = 37.48, P<0.001). The incidence rate of serological cure in the duration of follow-up was 6 cases per 100 per month (359/6282).

### Factors associated with serological cure

We analyzed the influencing factors associated with serological cure at 24 months in syphilis patients after therapy (Table 1). Males were more likely to have a serological cure than females (88.3% vs 80.0%, P<0.05). The patients who received tests actively presented an increased probability of serological cure compared with those who received passive test (87.5% vs 71.4%, P<0.05). Having a baseline TRUST titer >1:8 was associated with increased probability of serological cure compared with having a baseline TRUST titer ≤1:8 (93.6% vs 69.9%, P<0.05). Compared with participants with latent early syphilis, those with primary syphilis (93.0% vs 73.2%, P<0.001) and secondary syphilis (92.9% vs 73.2%, P<0.05) were associated with increased a probability of serological cure. A higher probability of serological cure was observed among homosexual patients (89.5% vs 85.3%, P<0.05), but not among bisexual patients. sex, age, marital status and treatment regimens were not associated with serological cure (P>0.05).

**Table 1:** Univariable and multivariate Cox proportional hazard analysis of the characteristics of patients who achieved serological cure.

	Univariable analysis			Multivariate analysis		
characteristics	Serological cure, no. (%) of participants (n = 418)	HR (95%CI)	P	characteristics	HR (95%CI)	P
**Sex**				**Baseline TRUST titer**		
female	96(80.0)	1		≤1:8	1	
male	263(88.3)	1.31(1.04,1.66)	0.02	>1:8	1.43(1.10,1.85)	<0.01
**Age**				**syphilis stages**		
15–19	12(100.0)	1		latent early syphilis	1	
20–29	160(85.6)	1.07(0.59,1.92)	0.83	primary syphilis	1.72(1.27,2.33)	<0.001
≥30	187(85.4)	1.04(0.58,1.86)	0.91	secondary syphilis	1.21(0.93,1.57)	0.15
**Marital status**						
unmarried	196(85.22)	1				
married	163(86.70)	0.84(0.68,1.03)	0.10			
**Detection**						
passive test	30(71.4)	1				
active test	329(87.5)	1.64(1.13,2.38)	0.01			
**Baseline TRUST titer**						
≤1:8	95(69.9)	1				
>1:8	264(93.6)	1.32(1.04,1.67)	0.02			
**Syphilis stages**						
latent early syphilis	109(73.2)	1				
primary syphilis	93(93.0)	1.78(1.35,2.35)	<0.001			
secondary syphilis	157(92.9)	1.40(1.10,1.79)	0.01			
**Treatment regimens**						
one course of BPG	336(85.9)	1.37(0.90,2.09)	0.14			
two courses of BPG	23(85.2)	1				
**Male Homosexuality**						
yes	51(89.5)	1.44(1.07,1.94)	0.02			
no	308(85.3)	1				
**Bisexual**						
yes	14(93.3)	1(0.59,1.71)	1.00			
no	345(85.6)	1				

A multivariate analysis was then conducted using all the related variables described above. The results (Table 1) further confirmed that having a baseline TRUST titer >1:8 was associated with an increased probability of serological cure, compared with having a baseline TRUST titer ≤1:8 (HR = 1.43, 95% CI = 1.10–1.85, P<0.01); primary syphilis (HR = 1.72, 95% CI = 1.27–2.33, P<0.001) was positively associated with serological cure, compared with participants with latent early syphilis.

## Discussion

The serological response has been widely used to evaluate the response to therapeutic regimens among syphilis patients [18]. Although previous studies have discussed the serological response to syphilis treatment and have reported the predictors of serological cure [12,14], the follow-up duration in these studies was relatively short and they did not consider the influence of time when analyzing the predictors of serological cure. In this study, 418 eligible patients were enrolled at the endpoint of 24 months and multivariate Cox proportional hazard analysis was performed to analyze the predictors of serological cure after treatment. Finally, 359 of the 418 early syphilis patients treated with BPG presented a serological cure after receiving a 2-year follow-up according to the TRUST test. The proportion of serological cures was 85.9%, higher than that reported by Sena AC [12] and Man-Li Tong [14]. In total, approximately 6 cases presented serological cures per 100 per month. To some extent, Syphilis Convergence Case-management Program might play some beneficial functions among early syphilis patients, and it is necessary to strengthen the measures of syphilis prevention and control. The results of this study regarding the response status and related factors first reported in Shenzhen will have implications for the further management of early syphilis and expected outcomes after therapy.

In this study, we found that sex, detection method and male homosexuality were associated with serological cure according to bivariate analysis, but not multivariate analysis. Although these variables did not have an independent function in the outcome, further study to explore this association is merited. Moreover, using multivariate analysis, we identified two potential factors associated with serological cure after treatment-baseline TRUST titer and disease phase.

Nontreponemal antibody titers are considered to be related to disease activity [19]. Romanowski, et al. reported that lower baseline titers (e.g., ≤ 1:8) in early syphilis were associated with an increased likelihood of seroreversion [20]. However, in our study we found that syphilis patients with higher baseline titers were more likely to achieve serological cure, which was the same as results from other studies [12,14]. A high baseline titer signifies a beneficial inflammatory and immune response to *Treponema pallidum*, which facilitates its clearance [21]. This outcome supported by an earlier study [22] that disclosed that Venereal Disease Research Laboratory (VDRL)-immunized rabbits exhibited partial protection against reinfection with Treponema pallidum. Therefore, higher baseline nontreponemal titers may indicate a beneficial inflammatory and immune response to *T*. *pallidum*.

In our study, a positive relationship between the stage of infection and serological cure was found. Specifically, compared with latent early syphilis, primary syphilis patients were more likely to achieve serological cure. A previous study also showed that primary syphilis patients had a higher rate of serological cure than secondary and latent syphilis patients [23]. This might be because the epidemiological history of patients with primary syphilis was relatively clear and could be treated in a timely manner. Commonly, the clinical staging of syphilis infection is different, and its symptoms are complicated. Therefore, in the progress of syphilis prevention and control, the syphilis stage should be considered a crucial factor for clinical treatment and centralized management.

There are several limitations to our study. First, there may be selection bias, and some participants were lost to follow-up. Second, some information bias may exist, especially in collecting private information since the participants may not have given accurate answers. Three, some variables (e.g., patients with a history of syphilis) were not included in the analysis because of the relatively small sample size.

## Conclusions

In conclusion, the present study showed a higher serological cure level in early syphilis patients after BPG therapy. Meanwhile, serological cure was significantly associated with the baseline serum titer and syphilis stage.

## Abbreviations

BPG: Benzathine penicillin G
TRUST: Toluidine red unheated serum test
HIV: Human immunodeficiency Virus
TPPA: Treponema pallidum particle agglutination test
HR: Hazard ratio
CI: Confidence interval
